# Unpowered Scooter Injury in Children at a Korea Level I Trauma Center

**DOI:** 10.3389/fped.2021.561654

**Published:** 2021-04-23

**Authors:** Min Ae Keum, Min Jeng Cho

**Affiliations:** Department of Surgery, Ulsan University Hospital, University of Ulsan College of Medicine, Ulsan, South Korea

**Keywords:** head injury, unpowered scooter, trauma, children, protective gear

## Abstract

**Purpose:** Unpowered scooters for recreation and transportation are popular among children. However, to date, there have been no studies on scooter-related injuries in Korea. This study aimed to assess the frequency and injury pattern with unpowered scooters and to propose prevention strategies.

**Methods:** Medical records of children aged <16 years with unpowered scooter-related injuries who visited the emergency department (ED) from 2007 to 2018 were retrospectively reviewed.

**Results:** A total of 109 children were included. The majority (78.9%) of injuries occurred during the last 3 years of the study. The mean age was 5.2 years, and 88% of children were <8 years of age. Most injuries (80.7%) occurred from a fall off a scooter. A total of 65.1% (*n* = 71) of injuries were to the head and face, followed by upper limb, lower limb, and torso injuries. Eight patients had an intracranial injury and skull fracture. Twenty children had limb fractures, and the most common site of fracture was the elbow. None of the patients wore any protective gear.

**Conclusions:** Unpowered scooter-related injuries are on the increase and represent a serious problem among younger children. The head and face, where serious injuries can occur, are the most vulnerable. Public and parental awareness and education regarding protective gear and safety guidelines are essential to prevent injuries.

## Introduction

Unpowered scooters are not only a popular form of childhood recreation but also have become one of the main choices for transportation because they are light, portable, and easy to handle. The enormous increase in scooter popularity has led to an increase in the number of reported scooter-related injuries (1–6). In 2001, 16 deaths were reported to the Consumer Product Safety Commission (CPSC)'s National Electronic Injury Surveillance System (NEISS) in the United States, 11 of which were in children (7). The CPSC reviewed the alarming injury data and recommended that children under the age of 8 years should not ride scooters without close adult supervision; children should not ride scooters on streets, near traffic, or in the dark; and children should wear helmets, knee pads, and elbow pads while riding a scooter (7, 8). Several studies have reported that wearing protective gear reduces injuries, and efforts are being made to change the perceptions of riders and parents through research and campaigns (9, 10). Nevertheless, the number of scooter-related injuries remains high (11, 12).

In Korea, unpowered scooters are called “kickboards” or “ssing-ssing car” and are particularly popular among preschool-aged children. Scooters are made of lightweight aluminum, with small low-friction wheels, a front handlebar, and a back-wheel brake. Two-wheeled scooters are most common in other countries and are popular with children of school age or older (1, 8, 13, 14). On the other hand, most of the popular scooters in Korea are equipped with three small wheels (two front wheels and one rear wheel). Scooter-related injuries have dramatically risen over the last few years in Korea, highlighted by the number of cases reported to the Consumer Injury Surveillance System (CISS) of the Korea Consumer Agency (KCA) of 184 in 2015 to 852 in 2019 (15). However, to date, there is no published literature describing scooter-related injuries in Korea.

This study reports the first series of children with unpowered scooter-related injuries in Korea. Our study aimed to describe the injury characteristics of children presenting with scooter injuries in a single institution over 12 years and to highlight the frequency and severity of the injury. We hope that this approach will help propose a prevention strategy and establish a legal basis in Korea.

## Materials and Methods

### Study Population and Clinical Data

All children aged <16 years with an unpowered scooter-related injury who visited the emergency department (ED) of Ulsan University Hospital between January 2007 and December 2018 were included in the study. Ulsan University Hospital is the largest hospital and the only level 1 trauma center in the Ulsan metropolitan area, with a yearly census of 2,500 visits of trauma patients aged <16 years.

Since there is no International Statistical Classification of Diseases (ICD)-10 code for scooter injuries, we manually screened trauma patients' ED registration lists and reviewed their medical records. All patient's medical records and radiographs were reviewed. We used a standardized trauma medical record form in the electronic medical record system. This form includes the time and places of the accident, mechanism of injury, and safety protective equipment. The diagnosis of a patient was based on the discharge code and the results of the radiological examination.

The injury severity score (ISS) was calculated as a measure of overall severity (16). The ISS is currently the most commonly used trauma score, which correlates with mortality, morbidity, and length of stay in the hospital. The ISS takes into consideration the precise definitive injuries found upon clinical evaluation, computed tomography (CT) or magnetic resonance imaging (MRI), or during operations. The ISS ranges from 0 to 75 and ISS > 15 is considered major trauma.

This study was conducted in accordance with the principles embodied in the Declaration of Helsinki, and ethical approval was granted by our hospital's Human Research Ethics Committee (IRB number: 2020-01-003). Acquisition of informed consent from study participants was waived owing to the retrospective nature of the study.

### Categorized Definition

The distribution and type of injuries on the body was categorized as follows: (1) head and face were classified into intracranial injury (ICI), isolated skull fracture, concussion symptoms without ICI, superficial injury including laceration/abrasion/bruise, and dental injury; (2) upper limb; (3) lower limb; and (4) torso were classified into the fracture, sprain, and superficial injury including laceration/abrasion/ bruise.

The places of the accident were categorized as follows: (1) public places (roads, sidewalks); (2) public recreational areas (parks, playgrounds); and (3) others (house, apartment hallway, staircase, etc.).

### Statistical Analysis

We used median and range, or mean ± standard deviation (continuous variables) and percentages (categorical variables) to describe the findings. We used linear by linear association to identify the annual trend of scooter injured patients. The proportion of the number of scooter injured patients was derived as the annual number of patients <16 years old with scooter injury per the respective year's trauma patients <16 years old visit to the ED. The data were analyzed using SPSS version 24 (IBM Corp., Armonk, NY, USA), and *P* < 0.05 indicated significant differences.

## Results

### Demographics

**Figure 1:**
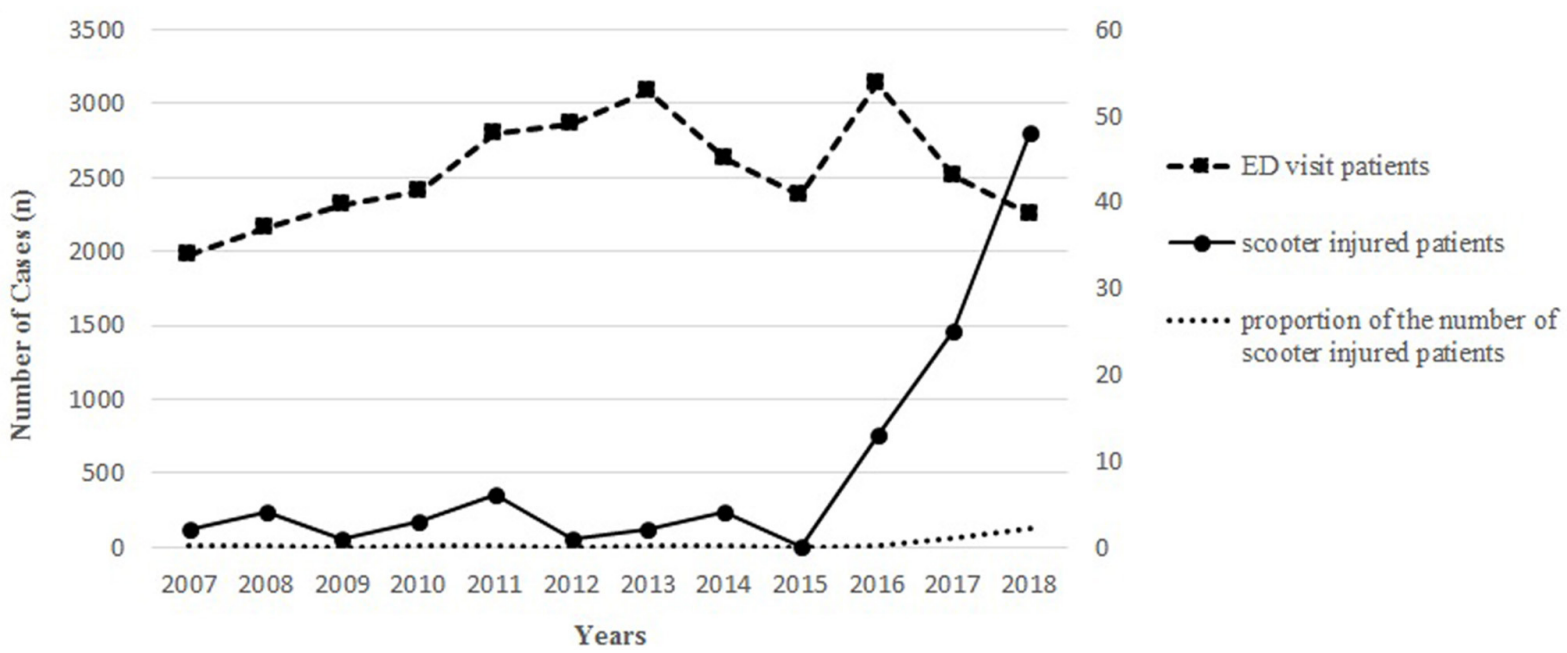
Number of unpowered scooter-related injury and the total number of ED visits from 2007 to 2018.

Altogether, 109 children were treated due to unpowered scooter-related injuries during the 12-year study period. As shown in Figure 1, scooter-related injuries significantly increased over the 12 years investigated (*P* < 0.001), and the majority (*n* = 86, 78.9%) of injuries occurred during the last 3 years of the study (Supplemental Table 1). Demographic data are presented in Table 1. The median age was 5 years (range, 17 months−13 years), and 65% of patients (*n* = 71) were male (Supplemental Figure 1). Ninety-six (88.1%) patients were <8 years old. The median ISS was 1 (range, 1–25). The ISS >15, which is considered major trauma was noted for six patients. Seventy-four (67.9%) injuries occurred from April to September. Radiological examinations were performed in all patients with X-ray, 51 CT, and three patients with MRI.

**Table 1:** Characteristics of unpowered scooter-related injuries.

**Characteristics**	**No. of cases, *n* = 109**
Age (years, mean ± SD)	5.2 ± 2.1
<8 years (%)	96 (88.1)
≥8 years (%)	13 (11.9)
Gender, male: female	71:38
Injury severity score (mean ±*SD*)	2.6 ± 4.2
Mechanism (%)	
Fall off a scooter	88 (80.7)
Fall off a staircase	1 (0.9)
Collision with a car^a^	9 (8.3)
Collision with a person	1 (0.9)
Collision with solid object^b^	4 (3.7)
Collision with bicycle	1 (0.9)
Handle or wheel injury	5 (4.6)
Place (%^c^)	
Public places	43 (59.7)
Public recreational areas	19 (26.4)
Others	10 (13.9)
Time (%)	
Daytime (6 a.m.−6 p.m.)	66 (60.6)
Nighttime (6 p.m.−6 a.m.)	43 (39.4)
Month (%)	
Jan.–Mar.	16 (14.7)
Apr.–Jun.	34 (31.2)
Jul.–Sept.	40 (36.7)
Oct.–Dec.	19 (17.4)
Weekday/Weekend (%)	
Weekday (Mon.–Fri.)	56 (51.4)
Weekend (Sat.–Sun.)	53 (48.6)
Protective gear worn (%)	0 (0)

a *Eight in road traffic accidents; one in a parking lot*.

b *One telegraph pole, one tree, two iron structures*.

c *Out of 72 patients*.

### Mechanism of Injuries and Place of Accidents

The majority (*n* = 88; 80.7%) of injuries occurred from a fall off a scooter. Fifteen patients (13.8%) were injured in collisions [car (*n* = 9), solid object (*n* = 4), another person (*n* = 1), bicycle (*n* = 1)] and one (0.9%) after falling down a staircase. Five patients (4.6%) were injured by the scooter handle or wheels.

The place of accidents was precisely recorded in 66.1% of the patients (*n* = 72). The most common place was public places such as roads and sidewalks (*n* = 43, 59.7%). Nineteen injuries (26.4%) occurred in public recreational areas, such as a park or playground. Ten (13.9%) occurred in other spaces: four in an apartment hallway and staircase, three in a house, two in a shopping center, and one in a parking lot. None of our patients wore any protective gear.

### Types and Distribution of Injuries

The types and distribution of injuries are summarized in Table 2. Of injuries, 65.1% (*n* = 71) were injuries to the head and face, followed by injuries to the upper limb (22.9%, *n* = 25), lower limb (7.3%, *n* = 8), and torso (4.9%, *n* = 5). Eight (11.3%) out of 71 head and face traumas were ICI and skull fracture (Table 3). All eight children were under 8 years of age. One had isolated skull fracture without ICI, and seven had dental trauma. Eighteen (25.4%) patients had no significant ICI or fracture but complained of concussion symptoms, including headache and vomiting. The other 37 (52.1%) patients had superficial injuries, such as laceration, abrasion, and bruise. Overall, patients with concussion symptoms, ICI, and skull fracture underwent head CT, and three patients underwent brain MRI. In patients with superficial injury, CT was performed in only 14 (37.8%) of 37 patients.

**Table 2:** Types and sites of injuries.

	***N* (%)**
Head and face (*n* = 71)	
Intracranial injury (ICI)^a^	8 (11.3)
Isolated skull fracture	1 (1.4)
Concussion symptoms without ICI on CT	18 (25.4)
Superficial injury^b^	37 (52.1)
Dental injury	7 (9.9)
Upper limb (*n* = 25)	
Fracture^c^	18 (72)
Superficial injury^b^	5 (20)
Sprain	2 (8)
Lower limb (*n* = 8)	
Fracture^d^	2 (25)
Sprain	6 (75)
Torso (*n* = 5)	
Superficial injury^e^	5 (100)

*ICI, intracranial injury; CT, computed tomography*.

a *all patients with intracranial injury had a skull fracture*.

b *laceration, abrasion, bruise*.

c *11 elbows, five wrists, one finger, and one scapula*.

d *two tibia/fibula*.

e *Chest area abrasion, shoulder pain, buttock laceration, labium majora laceration, and abdominal pain*.

**Table 3:** Eight patients with intracranial injury.

**Patient no**.	**Age/sex**	**Location**	**Mechanism**	**Injury details**	**ISS**	**Treatment**	**LOS (days)**
1	3/M	Park	Fall off	Parietal and temporal bone Fx SDH and EDH	25	Operation^a^	8
2	4/M	Unknown	Fall off	Parietal bone Fx Hemorrhagic contusion	9	Conservative	3
3	4/F	Unknown	Fall off	Parietal bone Fx, SDH	17	Conservative	6
4	5/M	Stairs	Fall off	Temporal bone Fx, EDH	16	Conservative	2
5	6/M	Park	Fall off	Occipital and temporal bone Fx, SDH	25	Conservative	7
6	7/M	Road	Collision with a car	Temporal bone Fx, SDH	9	Conservative	3
7	7/M	Road	Fall off	Parietal bone Fx, SDH	16	Conservative	3
8	7/M	Road	Fall off	Temporal bone Fx, EDH	16	Conservative	7

*ISS, injury severity score; LOS, length of hospital stay; Fx, fracture; SDH, subdural hematoma; EDH, epidural hematoma*.

a *Craniotomy, hematoma removal, and bleeding control*.

Eighteen (72%) of the upper limb injuries were fractures. The elbow was the most common site of fracture (*n* = 11), followed by the wrist (*n* = 5). All patients with elbow fractures were under 8 years of age, and four out of five patients with wrist fractures were over 8 years of age. Two (25%) of the patients with lower limb injury had ankle fractures. All patients with limb injury were examined by X-ray, and nine (27.3%, 9/33) patients underwent CT.

Five torso injury patients presented with superficial injury (one with chest area abrasion, one with shoulder pain, one with buttock laceration, one with labium majora laceration, and one with abdominal pain). There were no abnormal findings on X-ray in five patients, and the patient with abdominal pain did not show any injury to the abdominal cavity on CT.

### Management

Twenty (18.3%) of the presenting patients were admitted to the hospital, with eight (7.3%) requiring admission to the trauma intensive care unit. The median length of hospital stay of the 20 patients was 7 days (range, 2–12 days). Among eight patients with cerebral hemorrhage, one patient required craniotomy, whereas the remaining seven improved after conservative treatment. Limb fracture in 11 (55%) out of 20 patients required surgical treatment. The remaining children with fractures were conservatively treated with a cast. There was no complication or mortality.

## Discussion

This is the first study of unpowered scooter-related injuries in children in Korea. We found that the most distinctive feature of scooter injuries in Korea is the young age (mean age: 5.2 years). Many studies report an average age of 9–11 years, and to our knowledge, this study's mean age of 5.2 years is the youngest (9, 10, 17, 18). In 2001 NEISS data in the US showed that 85% of persons treated in the ED for unpowered scooter-related injury were children aged <15 years, and 23% were aged <8 years (7). Younger children have an underdeveloped neuromuscular system and higher center of gravity, which makes balancing more difficult and makes it easy for children to fall off the scooter. Moreover, young children's cognitive immaturity may make them unable to distinguish potential dangers, such as a busy street, downhill, or uneven roads. This led to the CPSC recommendation of an 8-year age minimum for scooter use. The results of this study, which indicated that 88% of children injured on scooters are younger than the CPSC recommended age limit, are very concerning.

In our study, the mechanism and pattern of injury corresponded with findings from other research, but there were more head and face injuries than in other studies (7, 8, 10, 18). Kubiak et al. have reported in their study that a decrease in head injuries was noted with increasing age, while the proportion of extremity injuries increased simultaneously (19). In this study, 65.1% of injuries were to the head and face; this higher percentage is explained by our study containing more young children than other studies. Although the majority of head and face trauma patients sustained superficial injuries (e.g., lacerations, abrasion, bruise), there is a potential for serious injury leading to disability or death (3, 20). In our cases, eight patients had a severe head injury (ICI and skull fracture), and one required a craniotomy. All patients with ICI were <8 years old. This shows that younger children are more vulnerable to head injuries, which is a powerful reason for scooters to have safety regulations for younger children.

In the present study, the most common fracture sites were upper limbs. Several studies report that the commonest fractures also involved the wrist; the mechanism of wrist injury is associated with falling with outstretched arms (21, 22). Elbow fracture was the most common in our study. Elbow fracture patients were all under 8 years old, whereas 4 out of 5 wrist fracture patients were over 8 years old. We think that older children fall with their arms outstretched, leading to wrist injuries, whereas elbow injuries in younger children are caused when they fall over the upper body.

The effectiveness of safety equipment on the head, elbow, and knee injuries in similar recreation activities (e.g., with in-line skates, skateboards, and bicycles) has been proven already (23, 24). Many studies show that wearing protective gear cannot prevent but can at least reduce the severity of injuries sustained (1, 3, 19, 21). Bressan et al. reported that not wearing a helmet resulted in an independent risk factor for intracranial injuries on CT in a study analyzing the head injury of a recreational vehicle (6). Our study showed that all patients with ICI had skull fractures. Skull fracture increases the risk of ICI, and this could be avoided only by wearing a helmet. There are studies that emphasize helmet use on scooters, but the proportion of helmet use on scooters is very low compared with other recreational activities (9, 17, 25). In addition, in Korea, there are no guidelines on safe practice and the role of appropriate safety equipment for unpowered scooters. None of our patients wore any protective gear. Legislation and parental awareness are most important in ensuring the use of protective gear. Parents of young children often have the false belief that, because of their age, they are at low risk of injury, reasoning that they travel at low speeds and are less likely to perform stunts (26). However, most injuries occur at relatively low speeds and even under close adult supervision. Continued parental education regarding the importance of protective gear is required. We further believe that the use of protective equipment, especially helmets, should be legally required for children riding scooters.

The place of injuries was recorded precisely in only 66.1% of the patients. Among those, 58% occurred on the roads and the sidewalks. This shows that although scooters are recreational, they are treated as a means of transportation. Scooter riding in public areas is exposed to sudden stops and unexpected circumstances. The small-wheel scooter design makes it difficult to maintain stability when vibrations caused by uneven ground are transmitted to the handlebar. Uneven ground and obstacles are harder to distinguish in the dark. Among children, scooters should only be used in public recreational areas, and nighttime use should be avoided.

In this study, 78.9% of injuries occurred in the last 3 years, showing a rapid increase in recent years. The cause of the recent rapid increase is unknown; we believe that scooters have become popular due to recreational trends and changing perceptions of scooters. The 2019 Child Safety Accident Trend Analysis of the KCA shows an increasing pattern similar to that of this study (15). According to the report, the scooter-related injuries among children <15 years old were 184 cases in 2015, 600 cases in 2017, and 852 cases in 2019, showing a rapid increase since 2016. Until 2017, bicycles were the most common cause of recreational vehicle-related injuries for children <15 years old; however, after 2018, the scooter injury was reported to be higher than bicycle injuries (48.1 vs. 38.8%, respectively). The incidence based on age was 24.9% in children aged between 1 and 3 years, 49.2% in 4 and 6 years, and 25.9% in 7 and 14 years, showing similar results to those of our study. The rapidly increasing number of injuries recently suggests that more research and safety guidelines for scooter-related injuries are needed.

Our single-center retrospective design has a few limitations, particularly with regard to the accuracy of data reporting and generalizability. First, limited documentation prevented us from identifying more detailed injury mechanisms and place, and factors influencing the occurrence of accidents such as weather, ground state, and slope. Furthermore, we could not identify adult supervision and the length of the scooter experience. Second, the diagnosis was based on discharge code and radiographic results, and the ED discharged patients were not followed-up. In patients who did not undergo CT, a missed injury may be present and delayed symptoms may have been overlooked. Finally, our data may not be generalizable to all children injured with scooters. Our center is an ED of a tertiary care center and the sole state trauma center in Ulsan. Severe injuries such as cerebral hemorrhage will be referred only to our hospital, but patients with mild injuries or fractures may visit other hospitals or outpatient clinics. Despite these limitations, this study demonstrates injury characteristics and trends for unpowered scooters in Korea and highlights an important emerging public health issue in children. We hope that it will help in facilitating the safe use of unpowered scooters and developing safety guidelines in the future.

In conclusion, this study shows that unpowered scooter-related injuries are on the increase and are particularly a serious problem among younger children. The head and face are the most vulnerable; these injuries are sometimes serious and require significant intervention. Children and parents should be aware of the potential dangers of scooter riding, and protective gear should be worn. Unpowered scooters should be restricted to use by children over 8 years and should not be used in hazardous environments such as on uneven grounds, on roads, or at night.

## Data Availability Statement

The raw data supporting the conclusions of this article will be made available by the authors, without undue reservation.

## Ethics Statement

The studies involving human participants were reviewed and approved by Ulsan University Hospital's Human Research Ethics Committee (IRB number: 2020-01-003). Written informed consent from the participants' legal guardian/next of kin was not required to participate in this study in accordance with the national legislation and the institutional requirements.

## Author Contributions

MC: conceptualization, formal analysis, validation, writing—original draft, and writing—review and editing. MK: investigation. MK and MC: Data curation, methodology, contributed to the article and approved the submitted version. All authors contributed to the article and approved the submitted version.

## Conflict of Interest

The authors declare that the research was conducted in the absence of any commercial or financial relationships that could be construed as a potential conflict of interest.

## Supplementary Material

Supplemental Figure 1 Age and sex distribution.

Supplemental Table 1 Number of unpowered scooter-related injury and the total number of ED visit, from 2007 to 2018.
