# Supplementary figures and images for: Unpowered Scooter Injury in Children at a Korea Level I Trauma Center
Source: Front Pediatr. 2021 Apr 23;9:561654. doi: 10.3389/fped.2021.561654 (PMC8102824; doi:10.3389/fped.2021.561654)

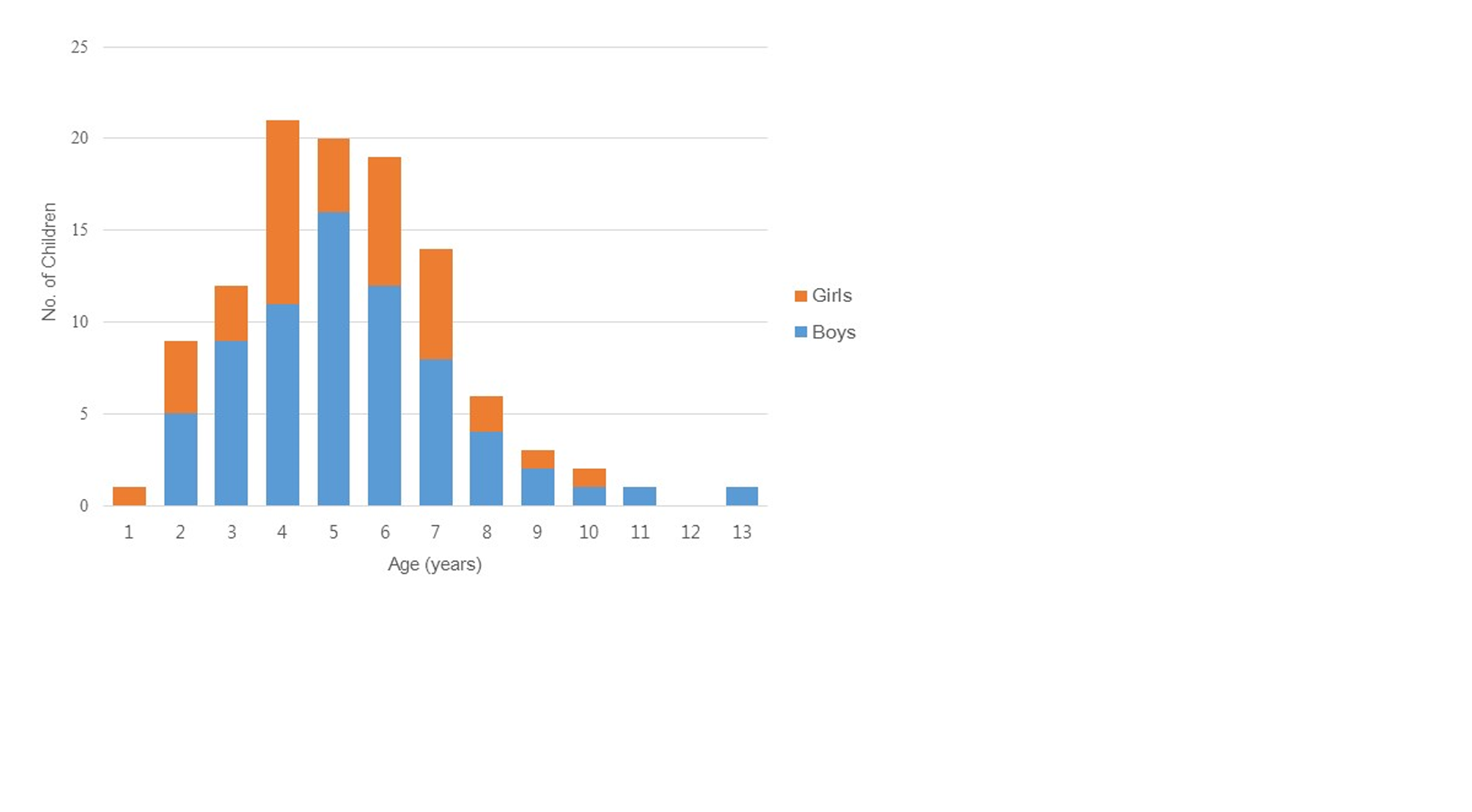

Supplement: Supplemental Figure 1 — Age and sex distribution. [file Image_1.TIF]
