# Supplementary material for: Unpowered Scooter Injury in Children at a Korea Level I Trauma Center
Source: Front Pediatr. 2021 Apr 23;9:561654. doi: 10.3389/fped.2021.561654 (PMC8102824; doi:10.3389/fped.2021.561654)
Supplement: Supplemental Table 1 — Number of unpowered scooter-related injury and the total number of ED visit, from 2007 to 2018. [file Table_1.DOCX]

| Year | ER visit patients (n) | Scooter injured patients (n) | Proportion of the number of scooter injured patients (%) |
| --- | --- | --- | --- |
| 2007 | 1980 | 2 | 0.1 |
| 2008 | 2160 | 4 | 0.19 |
| 2009 | 2316 | 1 | 0.04 |
| 2010 | 2407 | 3 | 0.12 |
| 2011 | 2803 | 6 | 0.21 |
| 2012 | 2860 | 1 | 0.03 |
| 2013 | 3091 | 2 | 0.06 |
| 2014 | 2632 | 4 | 0.15 |
| 2015 | 2380 | 0 | 0 |
| 2016 | 3137 | 13 | 0.41 |
| 2017 | 2506 | 25 | 1 |
| 2018 | 2252 | 48 | 2.13 |

Supplemental Table 1. Number of unpowered scooter-related injury and the total number of ED visits from 2007 to 2018
